# Disila- and digermabenzenes†

**DOI:** 10.1039/d1sc00130b

**Published:** 2021-03-26

**Authors:** Takahiro Sasamori

**Affiliations:** Division of Chemistry, Faculty of Pure and Applied Sciences, Tsukuba Research Center for Energy Materials Science (TREMS), University of Tsukuba 1-1-1 Tennodai Tsukuba Ibaraki 305-8571 Japan sasamori@chem.tsukuba.ac.jp

## Abstract

Reactions of isolable disilynes and digermynes with alkynes can result in the formation of the corresponding disila- (DSBs) and digermabenzenes (DGBs), wherein two carbon atoms of the benzene ring are replaced by silicon or germanium atoms. Detailed structural and spectroscopic analyses of these DSBs and DGBs have revealed that they exhibit considerable aromaticity, comparable to that of benzene. However, in contrast to the all-carbon system benzene, these DSBs and DGBs are highly reactive toward small molecules such as oxygen, hydrogen, 1,3-dienes, and water. During the investigation of their reactivity, we discovered that a 1,2-DGB works as a catalyst for the cyclotrimerization of arylalkynes, which provides access to the corresponding 1,2,4-triarylbenzenes. In this perspective article, our recent progress in the area of DSB and DGB chemistry is summarized.

## Abstract 2

Reactions of isolable disilynes and digermynes with alkynes can result in the formation of the corresponding disila- (DSBs) and digermabenzenes (DGBs), wherein two carbon atoms of the benzene ring are replaced by silicon or germanium atoms.

## Graphical abstract

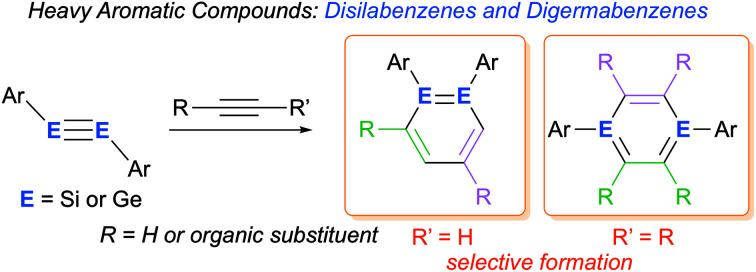

## 1. Introduction

**Fig. 1:**
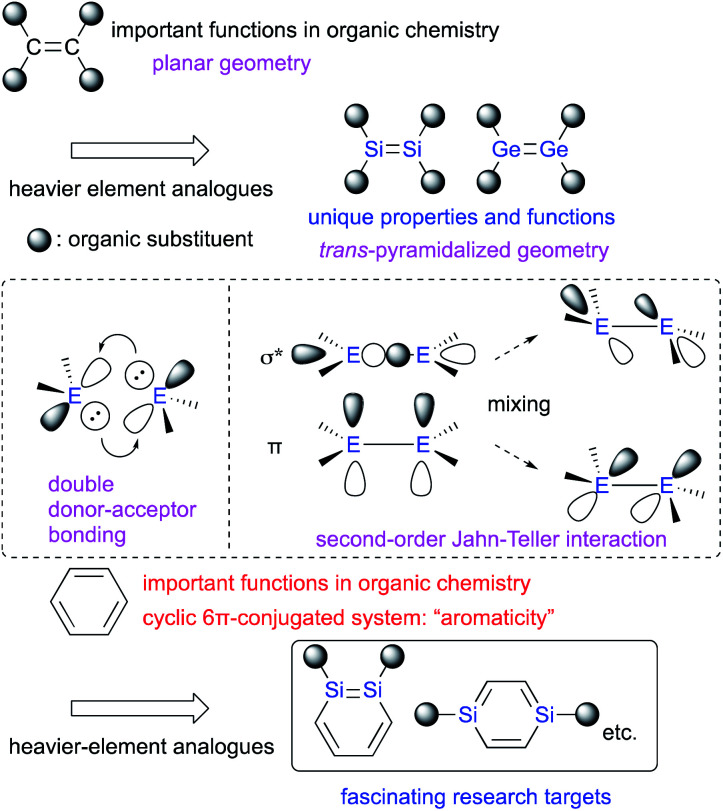
Heavier group-14 analogues of carbon-based π-bond compounds.

Multiple-bond compounds of heavier-group-14 elements represent the heavier homologues of unsaturated organic compounds.^1^ Traditionally, these compounds had been considered hard to isolate as stable monomeric compounds under ambient conditions. This notion was mostly due to their inherently high reactivity toward self-oligomerization and addition reactions with moisture and/or aerobic oxygen on account of their π-bonds, which are weak relative to the corresponding σ-bonds.^1,2^ However, it has since been demonstrated that the introduction of sterically demanding substituents on the heavier-group-14 elements can kinetically protect the corresponding π-bonds and thus render such compounds stable enough to be isolated under ambient conditions. Thus, several examples of kinetically stabilized aromatic compounds including a heavier-group-14 element using sterically demanding substituents have been reported,^1–3^ in addition to the already known heavy aromatic systems that bear a heavier-main-group element other than those from group 14 or a transition metal.^4^ With those examples in hand, the physical and chemical properties of the π-bonds between these heavier-group-14 elements have been thoroughly investigated experimentally and theoretically.^2^ It has been revealed that the bonding situation of these E=E double bonds are different from those of C=C double bonds. For example, in contrast to the planar geometry of most C=C double bonds, E=E bonds (E: heavier-group-14 element) often exhibit a *trans*-pyramidalyzed geometry. Moreover, the extent of bond-shortening of the E=E bond relative to the corresponding single bond is not as pronounced as for the C=C bonds. These structural features could be feasibly interpreted in terms of a double donor–acceptor bond (based on valence-bond theory) or so-called second-order Jahn–Teller mixing of the π- and σ*-orbitals (Fig. 1).^2,3^ Thus, in case of heavier-group-14 elements, the term “π-bond” would be formally suitable, as it comprises np-orbitals and the mixing of orbitals, which would be different from the carbon systems. In this regard, it should be of great interest, to replace not only a HC moiety of benzene with an RE moiety (R = substituent; E = heavier-group-14 element), but also a HC=CH moiety of benzene with an RE=ER moiety.

The concept of π-bonds is closely associated with the concept of ‘π-conjugation’, and both are of pivotal importance for not only fundamental aspects of organic chemistry, but also for materials science in order to create *e.g.* organic optoelectronic materials.^5^ In contemporary organic chemistry, benzene has received particular attention due to its characteristic ‘aromaticity’, which arises from its cyclic π-conjugation system in combination with the delocalization of its six π-electrons (Fig. 1).^6^ Benzene analogues that incorporate heavier-group-14 elements have also garnered much interest as potential building blocks for optoelectronic materials, albeit that some difficulties associated with isolating these compounds due to their intrinsic reactivity and/or facile self-oligomerization remain to be addressed.^6,7^ Recently, the synthesis and isolation of stable metallabenzenes that contain heavier-group-14 elements have been achieved by using bulky substituents, which allows evaluating the aromaticity and reactivity of the heavier-group-14-element analogues of benzene relative to that of the all-carbon homologue. In this context, the recent developments of our research on disila- (DSBs) and digermabenzenes (DGBs) will be summarized.

## 2. Theoretical aspects

**Fig. 2:**
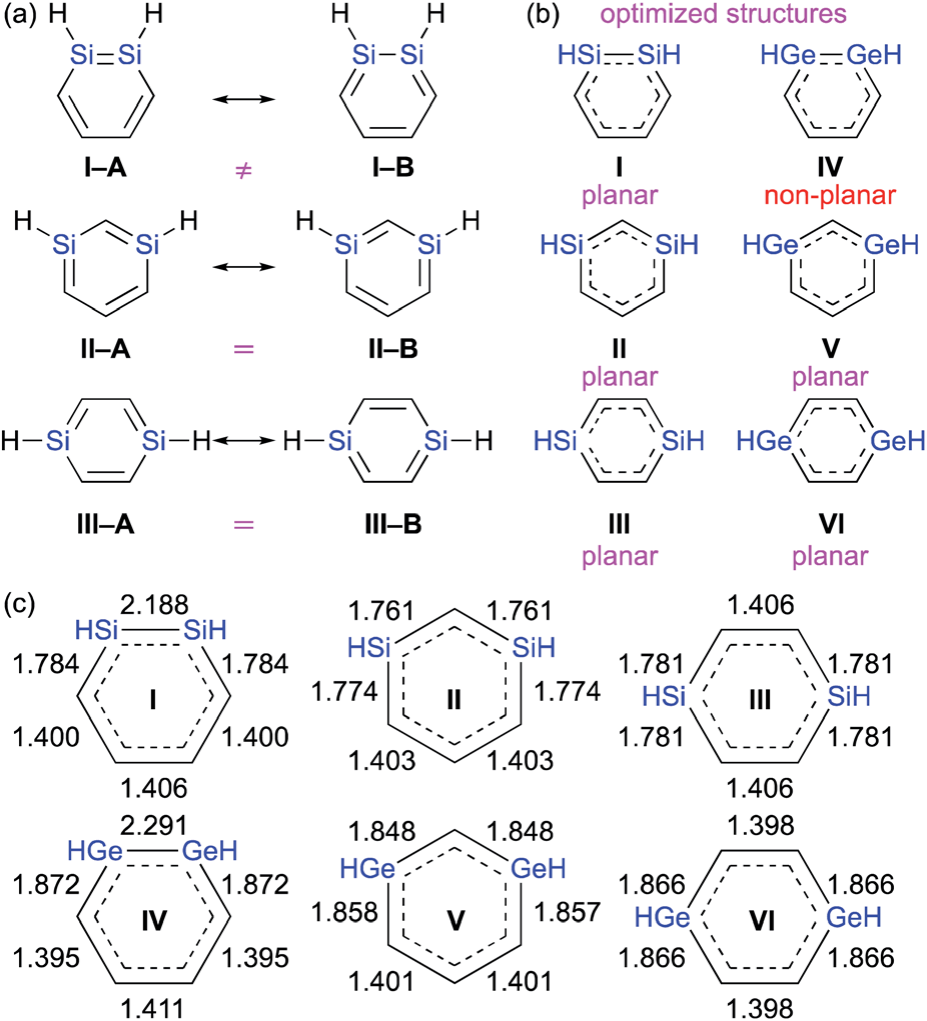
(a) Canonical resonance structures, (b) planarity of the optimized structures, and (c) optimized bond lengths of DSBs and DGBs, calculated at the MP2/6-311G(3d) level of theory.

Among the benzene analogues that contain two silicon or germanium atoms, 1,2-disilabenzenes (1,2-DSBs) or 1,2-digermabenzenes (1,2-DGBs) represent some of the most fascinating research targets, especially with respect to the effect of replacing a C=C unit in the 6π-electron aromatic system of benzene with an E=E unit (E = Si or Ge), considering the unique differences of the π-electron character of the disilene ( <svg xmlns="http://www.w3.org/2000/svg" version="1.0" width="10.400000pt" height="16.000000pt" viewBox="0 0 10.400000 16.000000" preserveAspectRatio="xMidYMid meet"><metadata> Created by potrace 1.16, written by Peter Selinger 2001-2019 </metadata><g transform="translate(1.000000,15.000000) scale(0.011667,-0.011667)" fill="currentColor" stroke="none"><path d="M80 1160 l0 -40 40 0 40 0 0 -40 0 -40 40 0 40 0 0 -40 0 -40 40 0 40 0 0 -40 0 -40 40 0 40 0 0 -40 0 -40 40 0 40 0 0 -40 0 -40 40 0 40 0 0 -40 0 -40 40 0 40 0 0 80 0 80 -40 0 -40 0 0 40 0 40 -40 0 -40 0 0 40 0 40 -40 0 -40 0 0 40 0 40 -40 0 -40 0 0 40 0 40 -40 0 -40 0 0 40 0 40 -80 0 -80 0 0 -40z M560 520 l0 -40 -40 0 -40 0 0 -40 0 -40 -40 0 -40 0 0 -40 0 -40 -40 0 -40 0 0 -40 0 -40 -40 0 -40 0 0 -40 0 -40 -40 0 -40 0 0 -40 0 -40 -40 0 -40 0 0 -40 0 -40 80 0 80 0 0 40 0 40 40 0 40 0 0 40 0 40 40 0 40 0 0 40 0 40 40 0 40 0 0 40 0 40 40 0 40 0 0 40 0 40 40 0 40 0 0 80 0 80 -40 0 -40 0 0 -40z"/></g></svg> Si=Si <svg xmlns="http://www.w3.org/2000/svg" version="1.0" width="10.400000pt" height="16.000000pt" viewBox="0 0 10.400000 16.000000" preserveAspectRatio="xMidYMid meet"><metadata> Created by potrace 1.16, written by Peter Selinger 2001-2019 </metadata><g transform="translate(1.000000,15.000000) scale(0.011667,-0.011667)" fill="currentColor" stroke="none"><path d="M480 1160 l0 -40 -40 0 -40 0 0 -40 0 -40 -40 0 -40 0 0 -40 0 -40 -40 0 -40 0 0 -40 0 -40 -40 0 -40 0 0 -40 0 -40 -40 0 -40 0 0 -80 0 -80 40 0 40 0 0 40 0 40 40 0 40 0 0 40 0 40 40 0 40 0 0 40 0 40 40 0 40 0 0 40 0 40 40 0 40 0 0 40 0 40 40 0 40 0 0 40 0 40 40 0 40 0 0 40 0 40 -80 0 -80 0 0 -40z M80 480 l0 -80 40 0 40 0 0 -40 0 -40 40 0 40 0 0 -40 0 -40 40 0 40 0 0 -40 0 -40 40 0 40 0 0 -40 0 -40 40 0 40 0 0 -40 0 -40 80 0 80 0 0 40 0 40 -40 0 -40 0 0 40 0 40 -40 0 -40 0 0 40 0 40 -40 0 -40 0 0 40 0 40 -40 0 -40 0 0 40 0 40 -40 0 -40 0 0 40 0 40 -40 0 -40 0 0 40 0 40 -40 0 -40 0 0 -80z"/></g></svg> ) moiety relative to the olefin moiety (C=C).^1–3,8^ In other words, it should be very interesting to investigate which one of the two conceivable canonical structures of 1,2-dimetallabenzenes (**I–A***vs.***I–B**) provides the predominant contribution to the overall electronic structure. This is different to the 1,3- and 1,4-dimetallabenzenes, where the conceivable canonical resonance structures **A** and **B** should exhibit the same electronic structures (Fig. 2).

**Fig. 3:**
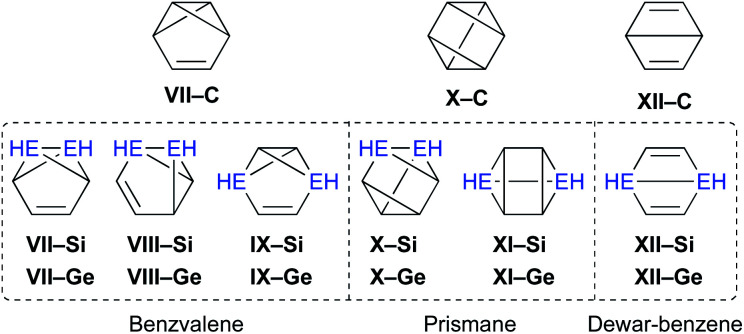
Possible valence isomers of benzene and dimetallabenzenes (E = C, Si, Ge) that bear no Si/Ge π-bond.

Moreover, the conceivable valence isomerizations of the dimetallabenzenes should also be very interesting (Fig. 3), given that the corresponding dimetalla-benzvalene, -prismane, and -Dewar-benzene isomers would exhibit unique cage structures. Considering that several theoretical investigations on dimetallabenzenes and their valence isomers have already been reported,^9^ we summarize only the most crucial results of our calculations in Table 1.^10^ Notably, 1,3-DSB is the most stable DSB, while 1,2-DGB is the most stable DGB. In addition, the relative energies of the valence isomers that do not contain an E=E double bond are shown in Table 1. As in the case of the all-carbon systems, the corresponding benzvalene-, prismane-, and Dewar-type isomers are thermodynamically unstable relative to the dimetallabenzenes, albeit that the energy difference between the valence isomers and the dimetallabenzenes is smaller than those for the corresponding carbon cases. Against this theoretical background, it seems feasible to assume that the synthesis of dimetallabenzenes, in contrast to some isolated valence isomers,^11^ remained unsuccessful for a long time due to the lack of appropriate synthetic routes and sufficient kinetic stability to avoid self-oligomerization.^12^ The unexpected thermodynamic stability of dimetallabenzenes relative to the other valence isomers should most likely be interpreted in terms of their considerable aromatic stabilization energy and/or the severe ring strain in the valence isomers.

Theoretical relative energies (kcal mol^−1^) of (a) dimetallabenzenes and (b) their valence isomers calculated at the MP2/6-311G(3d) level of theory (*E*_ZPE_, zero-point energy-corrected energies relative to that of benzene, 1,3-DSB, or 1,2-DGB)

**Table:** 

(a) Isomers	DSBs			DGBs		
	1,2-(**I**)	1,3-(**II**)	1,4-(**III**)	1,2-(**IV**)	1,3-(**V**)	1,4-(**VI**)
Relative energy	5.3	0.0	10.8	0.0	11.0	19.5

a Not the minimum structure.

**Table:** 

(b)	Benzvalene			Prismane		Dewar-benzene
E = C	**VII–C**			**X–C**		**XII–C**
Relative energy	74.6			118.1		81.0
E = Si	**VII–Si**	**VIII–Si**	**IX–Si**	**X–Si**	**XI–Si**	**XII–Si**
Relative energy	35.8	—a	22.3	58.5	69.1	26.6
E = Ge	**VII–Ge**	**VIII–Ge**	**IX–Ge**	**X–Ge**	**XI–Ge**	**XII–Ge**
Relative energy	—a	—a	40.6	59.1	68.1	29.3

The theoretically optimized bond lengths of DSBs and DGBs should be indicative of their resonance structures (Fig. 2). Even though the C–C bond lengths in 1,2-DSBs are almost identical, those in 1,2-DGBs are slightly different (1.395 and 1.411 Å), which suggests nonnegligible bond-alternation and a Ge=Ge character. Moreover, 1,2-DGB **IV** exhibits a non-planar geometry with a *trans*-bent structure at the Ge=Ge moiety, which is similar to other isolable digermenes,^8^ and different to the completely planar structures of other DSBs and DGBs. In their entirety, the results of our theoretical calculations show that the π-electrons of the Ge–Ge moiety in a 1,2-DGB should be localized to at least some extent in the cyclic 6π-electrons conjugation system.

**Fig. 4:**
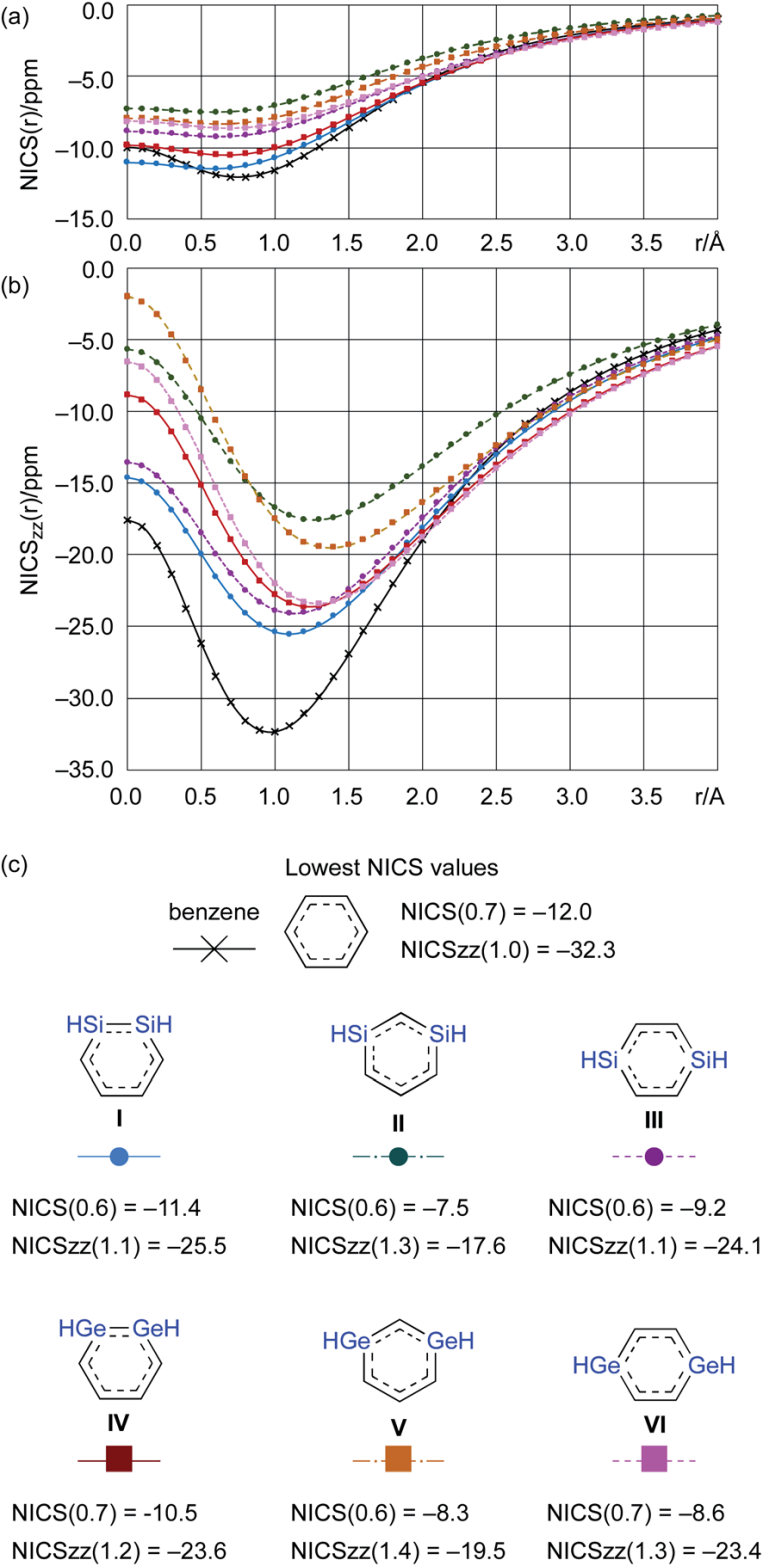
(a) NICS(*r*) plot, (b) NICS_zz_(*r*) plot, and (c) the lowest NICS values for benzene, DSBs (**I**, **II**, **III**), and DGBs (**IV**, **V**, **VI**), calculated at the GIAO-MP2/6-311G(3d)//MP2/6-311G(3d,p) level of theory.

From a theoretical perspective, several indicators have been proposed for the evaluation of aromaticity.^13^ In order to compare the aromaticity of benzene, DSBs, and DGBs, the NICS(*r*) and NICS_zz_(*r*) values for H-substituted model compounds have been calculated at the GIAO-MP2/6-311G(3d)//MP2/6-311G(3d) (Fig. 4).^14^ The NICS_zz_(*r*) profile for benzene exhibits the highest absolute value (−32.3) at *r* = 1.0 Å above the center of the C_6_ plane, while the highest absolute value (−12.0) for the corresponding NICS(*r*) profile is observed at *r* = 0.7 Å. The profiles for DSBs **I**, **II**, and **III** as well as DGBs **IV**, **V**, and **VI** are similar in as far as they show considerable aromaticity, even though their absolute values are slightly lower than those of benzene (Fig. 1c). Based on the NICS(*r*) and NICSzz(*r*) profiles for both DSBs and DGBs, the 1,3-dimetallabenzenes exhibit the lowest degree of aromaticity among the corresponding dimetallabenzene derivatives. Based on the highest absolute NICS(*r*) and NICSzz(*r*) values, it can thus be concluded that the aromaticity in the DSBs and DGBs should be considerable, albeit lower than in benzene.

Considering these theoretical investigations, it should also be very interesting to examine the electronic structures of 1,2-DSBs and 1,2-DGBs, especially with respect to their resonance structures.

## 3. 1,2-Disila- and 1,2-digermabenzenes

**Scheme 1:**
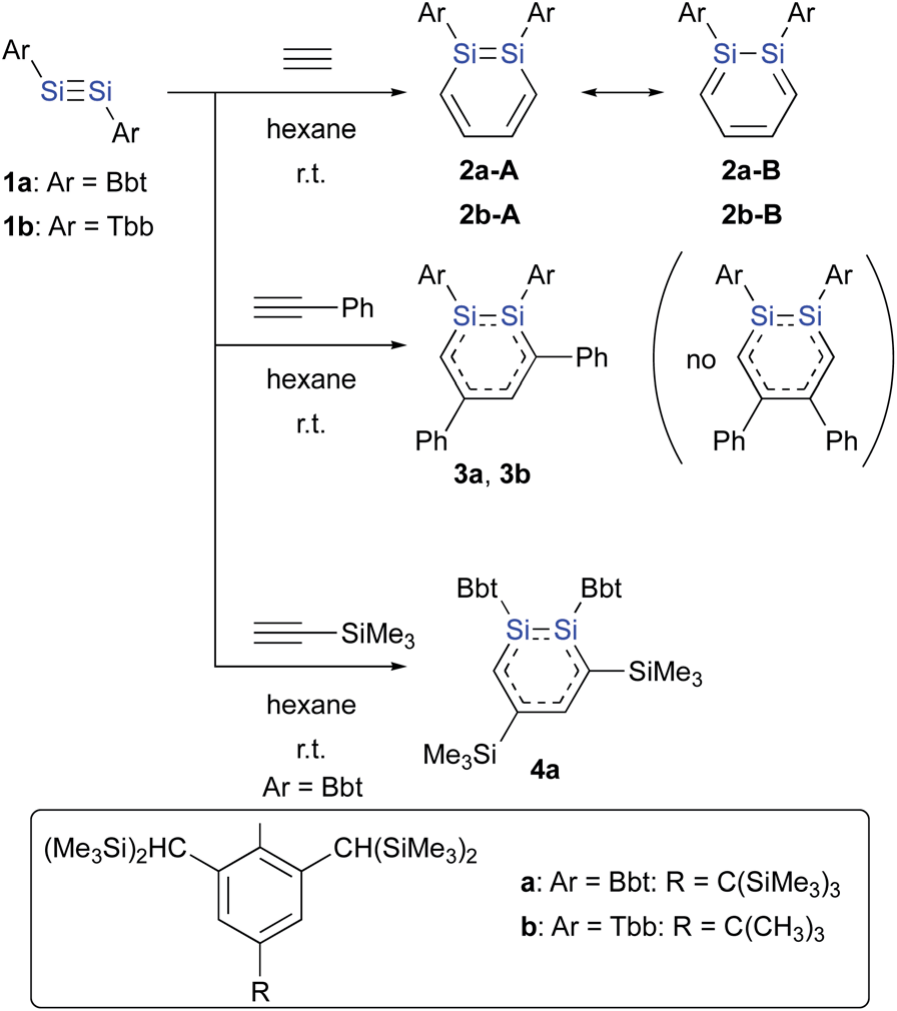
Formation of 1,2-DSBs.

The only hitherto reported synthetic route to 1,2-DSBs proceeds *via* the reaction of an isolable disilyne (RSi≡SiR)^15^ with acetylenes, *i.e.*, a formal [2 + 2 + 2] cycloaddition (Scheme 1).^16^ Sekiguchi *et al.* have reported the first successful isolation of a stable 1,2-DSB from the reaction of the stable disilyne R^Si^Si≡SiR^Si^ (**1c**; R^Si^ = Si(iPr)[CH(SiMe_3_)_2_]_2_)^15*b*^ with phenylacetylene.^16*a*^ However, the separation and purification of the two isomers obtained, *i.e.*, the 3,5-diphenyl- and 4,5-diphenyl-1,2-DSBs (3 : 2 ratio) were problematic due to the lability of the products toward air and moisture.

Given that stable diaryldisilynes ArSi≡SiAr (**1a**: Ar = Bbt; **1b**: Ar = Tbb; Bbt = 2,6-[(Me_3_Si)_2_CH]_2_-4-[(Me_3_Si)_3_C]-phenyl; Tbb = 4-*t*-butyl-2,6-[(Me_3_Si)_2_CH]_2_-phenyl) had already been reported by Tokitoh *et al.*,^17,18^ we wanted to use these as precursors for the synthesis of diaryl-substituted 1,2-DSBs.

The reactions of disilynes **1a** and **1b** with alkynes to give the corresponding 1,2-DSBs are shown in Scheme 1.^16*b*,18^ Treatment of hexane solutions of **1a** and **1b** with acetylene at r.t. afforded 1,2-Ar_2_-1,2-DSBs **2a** (Ar = Bbt) and **2b** (Ar = Tbb), respectively.^16*b*^ Thus, the substituent effect of Bbt and Tbb during the formation of 1,2-DSBs is negligible.^18^ In contrast to the reaction of disilyldisilyne **1c**,^16*a*^ the reaction of disilyne **1a** with phenylacetylene results in the selective formation of 3,5-diphenyl-1,2-DSB **3a** together with an inseparable and unidentified by-product, which was not the corresponding 4,5-diphenyl-1,2-disilabenzene. Conversely, treatment of **1b** with phenylacetylene in hexane at r.t. quantitatively afforded 3,5-diphenyl-1,2-DSB **3b** as the sole product.

**Scheme 2:**
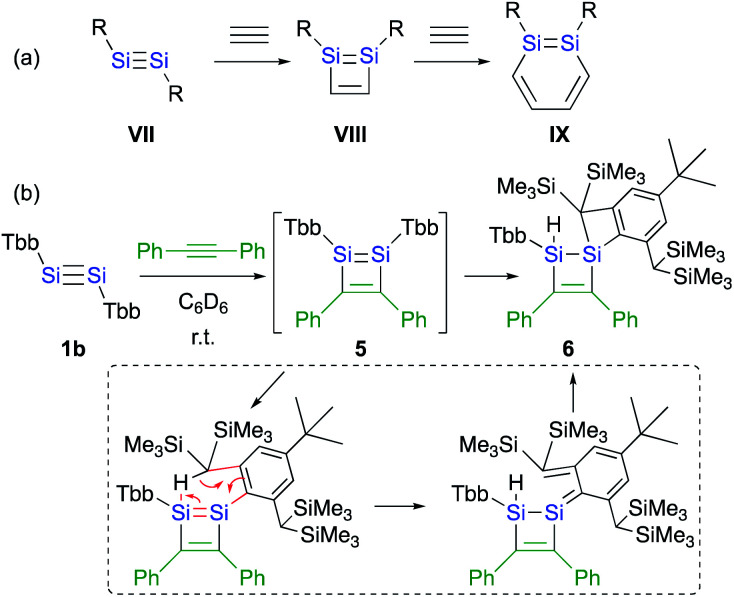
(a) A general scheme of a plausible formation mechanism for DSBs. (b) Reaction of disilyne **1b** with diphenylacetylene.

**Scheme 3:**
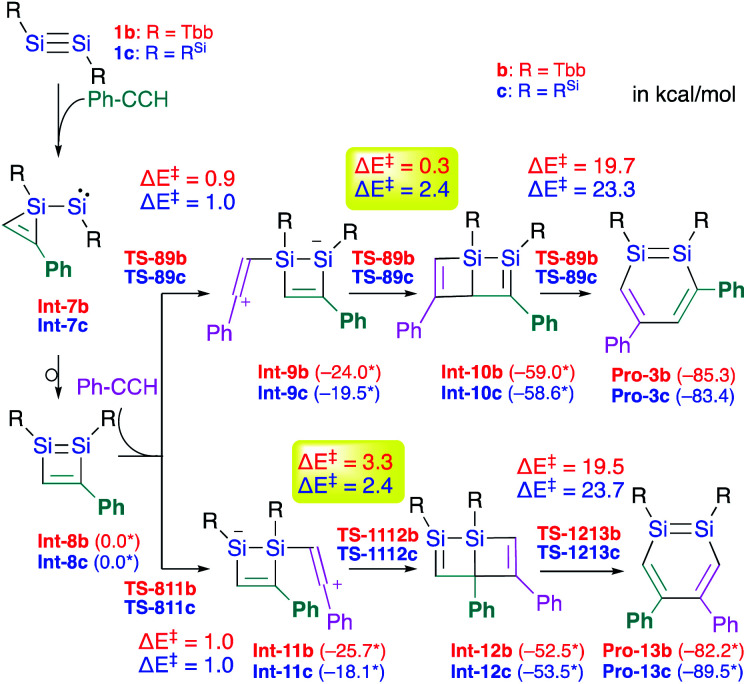
Theoretically investigated reaction mechanisms for the formation of 3,5-diphenyl- and 4,5-diphenyl-1,2-DSBs (**Pro 3** and **Pro-13**) with Tbb and R^Si^ substituents, together with the reaction barriers (in kcal mol^−1^) calculated at the B3PW91-D3(BJ)/lanl2dz+d (for Si) 6-31G(d) (for C,H) level of theory. *values in parentheses represent potential energies relative to that of **Int-8**.^18^

The difference with respect to the observed regioselectivity in the reaction of disilyldisilyne **1c** and diaryldisilyne **1b** with phenylacetylene is quite remarkable. The formation mechanism of a 1,2-DSB *via* the reaction of a disilyne with two molecules of acetylene has been theoretically investigated:^18^ a formal [2 + 2] cycloaddition between the Si≡Si moiety of the disilyne (**VII**) and the C≡C moiety of an acetylene results in the formation of the initial 1,2-disilacyclobutadiene intermediate (**VIII**), which can undergo a further reaction with another molecule of acetylene to give 1,2-disilabenzene **IX** (Scheme 2a). Based on this mechanism, the reaction of disilyne **1** with diphenylacetylene has been attempted in order to try and isolate the possible disilacyclobutene intermediate,^19^ similar to the case of the digermacyclobutadiene derivative (*vide infra*).^20,21^ When **1** was treated with diphenylacetylene, cyclic compound **6** was obtained quantitatively, suggesting the intermediate formation of 1,2-disilacyclobutadiene **5**, which would readily undergo a [1,5]-sigmatropic hydrogen migration followed by an intramolecular [2 + 2] cycloaddition (Scheme 2). Thus, as shown in previously reported theoretical investigations, the initial formal [2 + 2] cycloadditions of disilynes **1b** and **1c** with phenylacetylene to give the corresponding 3-phenyl-1,2-disilacyclobutadienes **Int-8b** and **Int-8c** could occur *via* the electrophilic attack of the Si≡Si moieties at the C≡C moieties, followed by an intramolecular ring expansion in **Int-7b** and **Int-7c** (Scheme 3). Moreover, the potential energy surfaces (PESs) of the subsequent reactions of 1,2-disilabutadienes **Int-8** with phenylacetylene to give the corresponding 1,2-DSBs have been theoretically investigated. Specifically, **Pro-3** and **Pro-13**, have been theoretically calculated for Tbb- and R^Si^-substituted models (Scheme 3).^18^

The insertion of the C≡C moiety of phenylacetylene into the Si–C bond of **Int-8** should be crucial for the selectivity of the 1,2-DSBs. In both cases (**b**: R = Tbb; **c**: R^Si^), the insertion of phenylacetylene into the 1,4-Si–C bond of **Int-8** would give 3,5-diphenyl-1,2-DSBs (**Pro-3**), while that into the 2,3-Si–C bond would lead to the formation of 4,5-diphenyl-1,2-DSBs (**Pro-13**). The entire process consists of (i) the addition of PhCCH (**Int-8** → **Int-9** or **Int-11**), (ii) a ring-closure (**Int-9** → **Int-10** or **Int-11** → **Int-12**), and (iii) a ring-expansion (**Int-10** → **Pro-3** or **Int-12** → **Pro-13**), whereby step (iii) should be the rate-determining step for the formation of **Pro-3** or **Pro-13**. However, the selectivity cannot be readily rationalized exclusively in terms of the reaction barriers of the rate-determining steps, which are almost identical (**b**: R = Tbb, Δ*E*^‡^ = 19.7 kcal mol^−1^ for **Pro-3b** and 19.5 kcal mol^−1^ for **Pro-13b**; **c**: R = R^Si^, Δ*E*^‡^ = 23.3 kcal mol^−1^ for **Pro-3c** and 23.7 kcal mol^−1^ for **Pro-13c**). In step (ii), the barriers for **Int-9c** → **Int-10c** and **Int-11c** → **Int-12c** are almost identical (Δ*E*^‡^ = 2.4 kcal mol^−1^), while that for **Int-9b** → **Int-10b** (Δ*E*^‡^ = 0.3 kcal mol^−1^) is by 3.0 kcal mol^−1^ lower than that for **Int-11b** → **Int-12b** (Δ*E*^‡^ = 3.3 kcal mol^−1^), which would explain the selective formation of **Pro-3b** in the reaction of **1b** with phenylacetylene in the Tbb-substituted case but not in the R^Si^-substituted case.

**Scheme 4:**
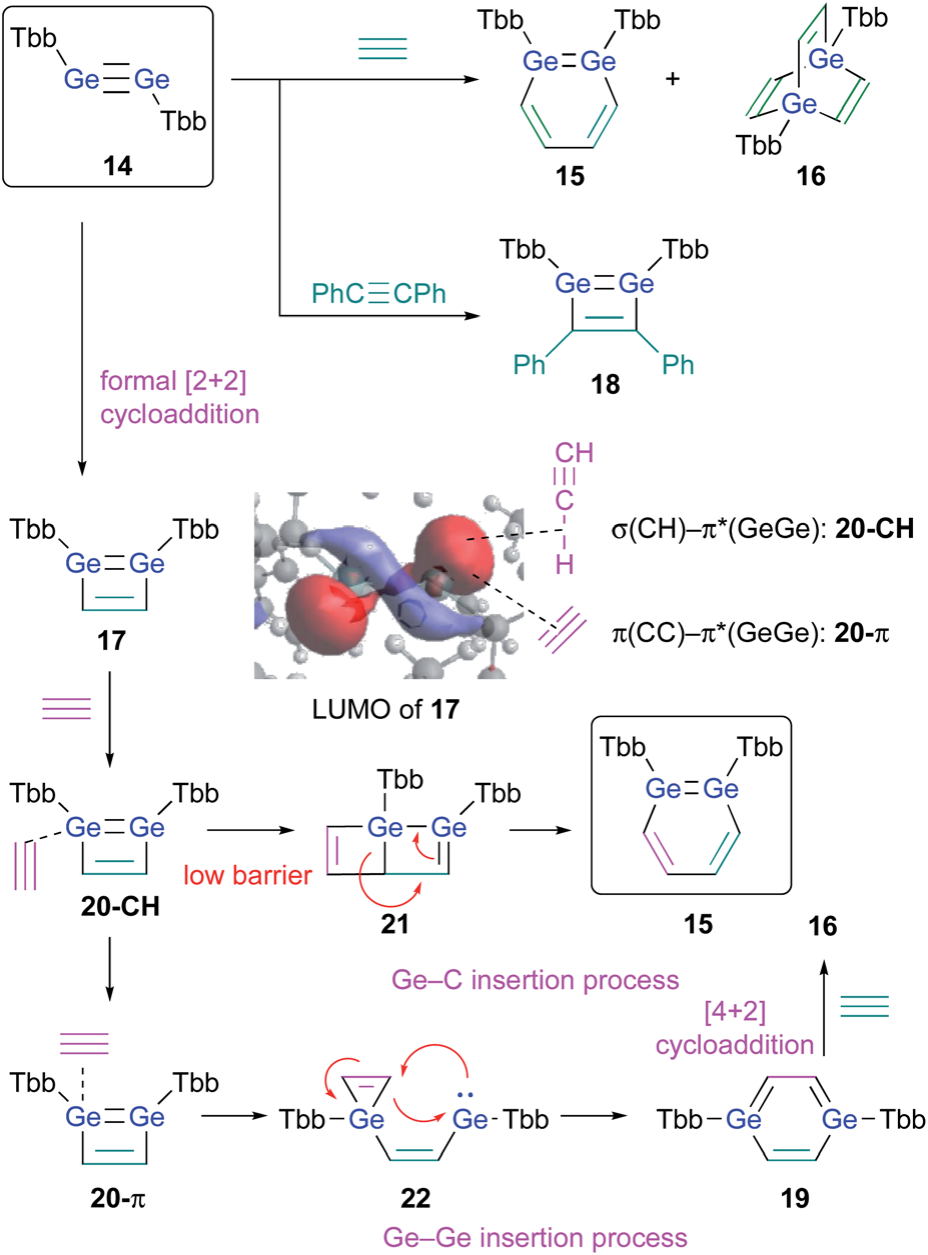
Formation of 1,2-DGB **15** and 1,4-digermabarrelene **16** in the reaction of digermyne **14** with acetylene.

The successful isolation of stable 1,2-DSBs from the reaction of the corresponding disilyne with alkynes^16,18^ naturally prompted us to examine the reactions with Ge-analogues, *i.e.*, the reaction of isolable digermynes with alkynes in the expectation to obtain the corresponding 1,2-DGBs.^20,22^ Digermyne TbbGe≡GeTbb (**14**) was prepared according to a previously reported synthetic procedure for stable digermynes.^23^ Exposure of a hexane solution of **14** to an acetylene atmosphere at r.t. afforded 1,2-DGB **15** (61% ^1^H NMR yield) as a pale yellow crystalline solid together with 1,4-digermabarrelene **16** (22% ^1^H NMR yield) (Scheme 4).^20^ Detailed theoretical calculations revealed that the formation of 1,2-DGB **15** should proceed according to a formation mechanism analogous to that for 1,2-DSBs **2a,b**, *via* the 1,2-digermacyclobutadiene intermediate **17**, which was experimentally supported by the formation of isolable 3,4-diphenyl-1,2-digermacyclobutadiene **18** in the reaction of **14** with diphenylacetylene.^20,21^ The formation of **16** could potentially be rationalized in terms of the intermediacy of 1,4-digermabenzene **19**, which can be expected to readily undergo a subsequent [4 + 2] cycloaddition with one molecule of acetylene to give **16**.

The potentially underlying reaction mechanism has been investigated using detailed DFT calculations.^20^ The key point for the selectivity between products **15** and **16** should be the interaction modes between **17** and acetylene. Given the characteristically low-lying π*(Ge=Ge) orbital of **17**, the σ orbital (CH) of acetylene would be approaching to form complex **20-CH** as an initial intermediate. **20-CH** would then be converted to complex **20-π**, which exhibits an orbital interaction between the C≡C π orbital and the π*(Ge=Ge) orbital, which is a mildly endothermic process. Conversely, with a slightly lower barrier relative to the conversion process to **20-π**, **20-CH** could undergo a pericyclic reaction to give **21**, which would furnish 1,2-DGB **15**. Complex **20-π** could be converted into 1,4-digermabenzene **19***via* the process shown in Scheme 4, and **19** could undergo a facile [4 + 2] cycloaddition with another molecule of acetylene to furnish **16**, which should be a thermodynamically very stable product, *via* a barrierless process. Thus, given all the reactions of **17** with acetylene, the pathways to the formation of 1,2-DGB **15** and 1,4-digermabarrelene **16** could be considered as kinetically and thermodynamically controlled processes, respectively.^20^

**Fig. 5:**
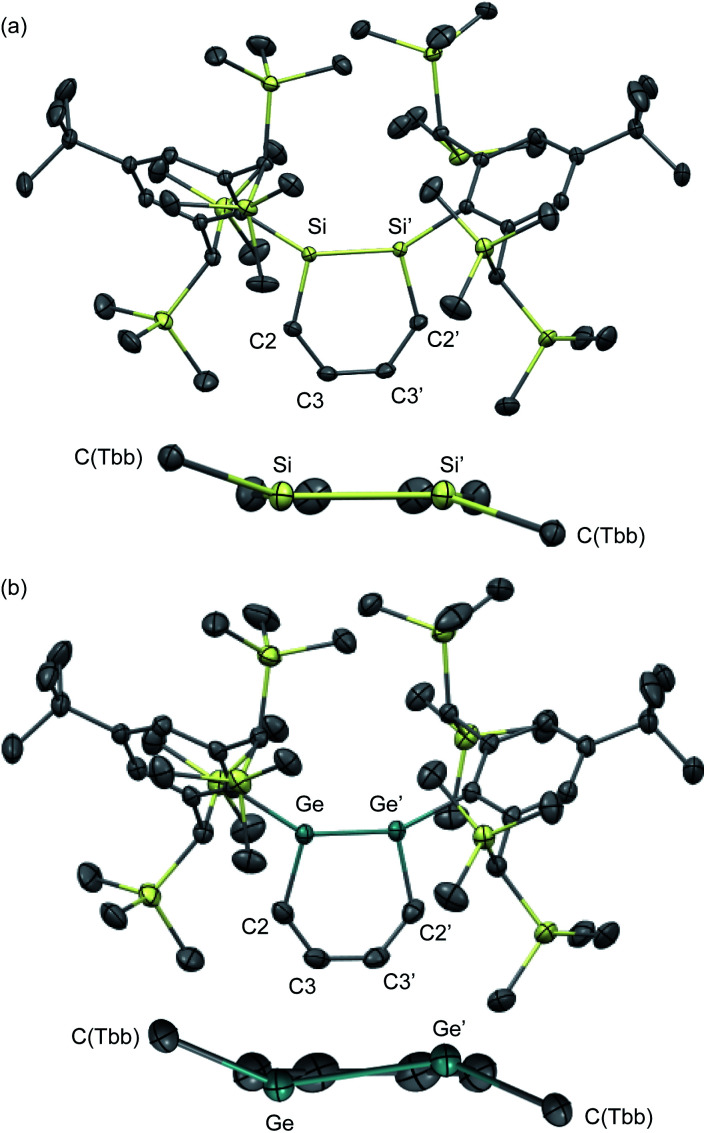
Molecular structures of (a) 1,2-DSB **2b** and (b) 1,2-DGB **15** with thermal ellipsoids at 50% probability; hydrogen atoms are omitted for clarity.

The molecular structures of 1,2-DSB **2b** and 1,2-DGB **15** are shown in Fig. 5,^18,22^ and selected structural parameters are summarized in Table 2. In both cases, the E_2_C_4_ moiety exhibits crystallographic C2 symmetry. Interestingly, the sum of the interior angles of the E_2_C_4_ rings (Table 2) suggests that the Ge_2_C_4_ ring of **15** adopts a non-planar geometry, wherein the Ge–Ge axis comprises an angle of *ca.* 8.6° relative to the C_4_ plane, which stands in sharp contrast to the planarity observed for the Si_2_C_4_ ring in **2b**.^17,18^ These structural features were expected considering the theoretical calculations shown in Fig. 2. The bond lengths in the E_2_C_4_ moieties (E–E′, E–C2, C2–C3, and C3–C3′) fall in between those of the corresponding single and double bonds. Thus, the six π electrons should be delocalized over the E_2_C_4_ rings, which suggests considerable aromaticity. However, the C3–C3′ bond lengths are slightly longer than the C2–C3 bonds in both cases, which indicates a predominant contribution from resonance structure **A** (Fig. 2) with E = E-double-bond character, rather than from resonance structure **B** with E–E single bond character.

Selected structural parameters for the solid-state structures of 1,2-DSB **2b** and 1,2-DBG **15**

**Table:** 

	Bond lengths/Å			
	E–E′	E–C2	C2–C3	C3–C3′
**2b**	2.2101(8)	1.8071(5)	1.3787(7)	1.4141(9)
**15**	2.3118(4)	1.897(3)	1.359(4)	1.417(4)

a Sum of the interior angles in the E_2_C_4_ moiety.

**Table:** 

	Bond angles/°			
	E′–E–C2	E–C2–C3	C2–C3–C3′	Σ_(E_2_C_4_)_^a^
**2b**	103.44(3)	130.10(4)	126.38(5)	719.8
**15**	101.34(9)	129.3(2)	128.1(3)	717.5

**Scheme 5:**
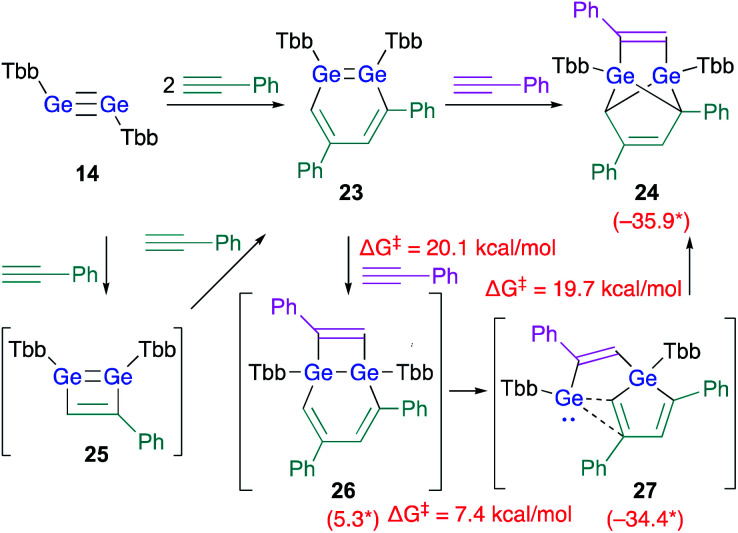
Reactions of digermyne **14** with phenylacetylene. *values in parenthesis represent free-energy values relative to that of **23** (in kcal mol^−1^); calculated at the TPSSTPSS-D3 level of theory.^24^

As described above, the reaction of disilyne **1b** with phenylacetylene selectively affords 3,5-diphenyl-1,2-DSB **3b**.^18^ As expected, the reaction between digermyne **14** and 2 equiv. of phenylacetylene at r.t. in C_6_D_6_ afforded 3,5-diphenyl-1,2-DGB as the sole product in 68% isolated yield.^24^ However, in contrast to the silicon case, 1,2-DGB **23** is able to undergo a further reaction with phenylacetylene at r.t. to furnish tricyclic **24** (Scheme 5).^24^

DFT calculations on the reaction mechanism of this reaction revealed the most reasonable pathway for the formation of **24** from digermyne **14**. The formation of 3,5-diphenyl-1,2-DGB **23** from **14** could be explained reasonably using 1,2-digermacyclobutadiene intermediate **25** in analogy to the afore-mentioned cases (*vide supra*). A third molecule of phenylacetylene could attack the LUMO of **23**, which should be predominantly localized around the Ge=Ge moiety, to give **26***via* a formal [2 + 2] cycloaddition. Then, the ring-contraction of the Ge_2_C_4_ moiety could occur *via* a carbon migration from one Ge atom to the other Ge atom to form germole–germylene **27**. Finally, an intramolecular [4 + 1] cycloaddition between the germole and the germylene moieties in **27** could afford **24**. Based on theoretical calculations at the TPSSTPSS-D3 level of theory, the rate-determining step for the entire processes should be **23** → **26** (Δ*G*^‡^ = 20.1; Δ*G* = 5.3 kcal mol^−1^).

**Scheme 6:**
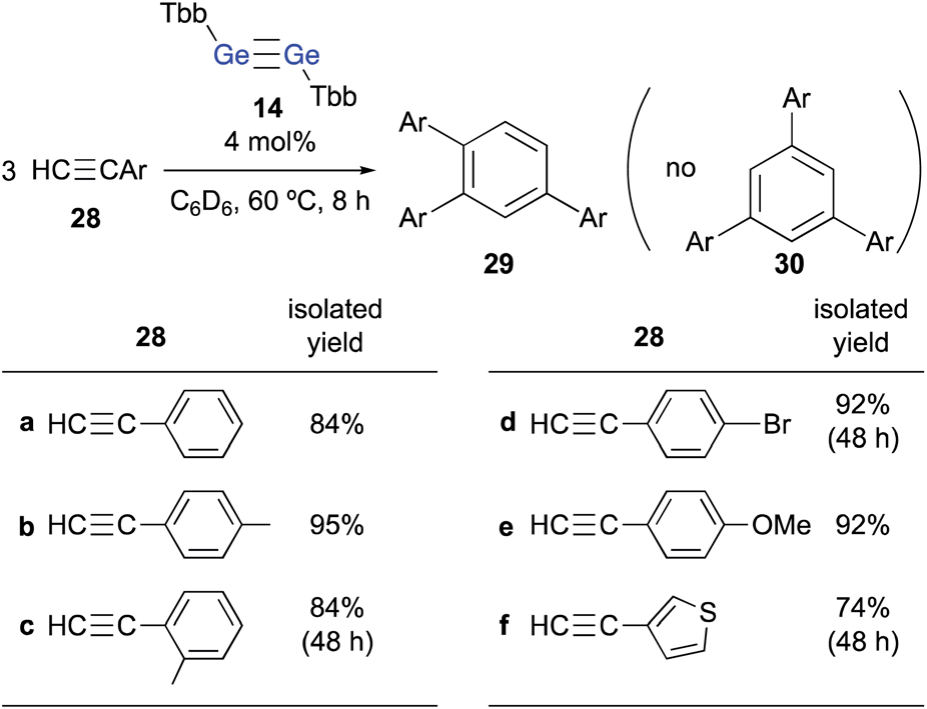
Cyclotrimerization of arylacetylenes **28** in the presence of a catalytic amount of digermyne **14**.

When **24** was further treated with phenylacetylene in C_6_D_6_ at 60 °C, phenylacetylene was selectively converted into 1,2,4-triphenylbenzene, while **24** was not consumed during the reaction. Given that simply heating phenylacetylene in the absence of any other compound under otherwise identical conditions did not show any reaction, it is feasible to conclude that **23** catalyzes the cyclotrimerization of phenylacetylene to give 1,2,4-triphenylbenzene. These experimental results demonstrate that the thermal reaction of arylacetylenes **28** in the presence of a catalytic amount of digermyne **14** smoothly furnishes the corresponding 1,2,4-triarylbenzenes (**29**) as the sole product in high yield (Scheme 6).^24^ Notably, the reaction proceeds with absolute stereoselectivity, *i.e.*, other triarylbenzene isomers such as **30** were not obtained in these reactions. Compound **24** should be the resting state of the catalyst, considering that it is the only compound that was observed during the ^1^H NMR monitoring of the catalytic reactions. Thus, digermyne **14** acts as a pre-catalyst for the cyclotrimerization of arylacetylenes in the absence of a transition-metal catalyst,^25^ specifically for the perfectly regioselective cyclotrimerization of terminal arylacetylenes.

**Scheme 7:**
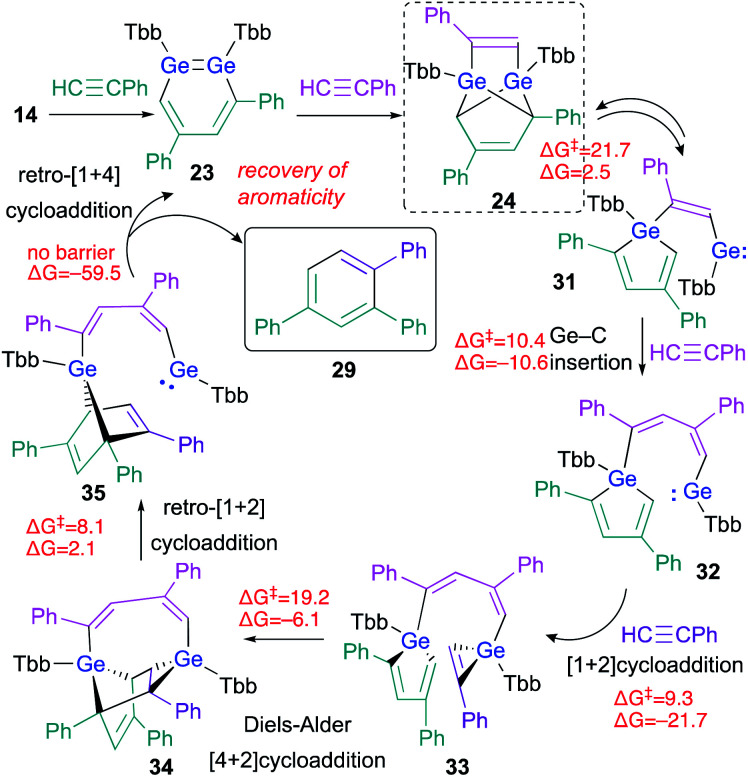
Plausible mechanism for the cyclotrimerization of phenylacetylene catalyzed by 1,2-DGB **23**; free-energy values are shown in kcal mol^−1^.

The experimental results, including crossover reactions, combined with the results of detailed theoretical calculations, have helped to reveal the reaction mechanism underlying the cyclotrimerization of phenylacetylene catalyzed by **23** (Scheme 7). The reaction barrier from **24** to **31** is the highest (Δ*G*^‡^ = 21.7, Δ*G* = 2.5 kcal mol; calculated at the TPSSTPSS-D3 level of theory) in the entire catalytic cycle shown in Scheme 7, which is consistent with the experimental result that only **24** was observed during the ^1^H NMR monitoring of the reactions.

As described above, the tricyclic skeleton of **24** can be considered as the intramolecular [1 + 4] cycloadduct of a germole and a germylene. Thus, the retro [1 + 4] cycloaddition of **24** should give germole–germylene **31** in the equilibrium state. Then, the Ge–C insertion of phenylacetylene could occur to give **32**, the germylene moiety of which could undergo a further [1 + 2] cycloaddition with phenylacetylene to give **33**.^26^ The intramolecular Diels–Alder reaction of **33** can then be expected to smoothly afford **34**. The ring-strain in **34** should be released *via* a retro [1 + 2] cycloaddition in the germirane moiety to furnish **35**. The driving force for the recovery of the aromaticity of both triphenylbenzene **29** and 1,2-DGB **23** would promote the conversion of germanorbornadiene **35** to **23** with concomitant elimination of **29***via* a retro [1 + 4] cycloaddition. The key step for the stereoselectivity in these reactions should be **33** → **34**, given the favorable orientations of the frontier orbital interactions, which are required for Diels–Alder reactions.^27^

In this section, we have discussed the synthesis of 1,2-DSBs and 1,2-DGBs, and plausible mechanisms of their formation have been postulated based on a combination of experimental and theoretical results. For the formation of both 1,2-DSBs and 1,2-DGBs, the key intermediates should be the corresponding 1,2-disila- and 1,2-digermacyclobutadienes. The effective orbital interaction between the σ(H–C≡) orbital and the low-lying LUMO of the 1,2-dimetallacyclobutadiene intermediates should promote the formation of the 1,2-dimetallabenzenes. Interestingly, digermyne **14** works as an effective pre-catalyst for the cyclotrimerization of arylacetylenes to selectively afford the corresponding 1,2,4-triarylbenzenes; in this process, the 1,2-DGB could be a key intermediate.

## 4. 1,4-Disila- and 1,4-digermabenzenes

Considering the aforementioned theoretical calculations on the reaction of digermyne **14** with acetylene (Scheme 4), which furnishes 1,2-digermabenzene **15** and 1,4-digermabarrelene **16**, it has suggested that the orbital interaction between the π*(Ge=Ge) orbital of **17** and the σ(H–C≡) orbital of an alkyne would lead to the formation of 1,2-DGB **15**, while that between the π*(Ge=Ge) orbital of **17** and the π(C≡C) orbital of an alkyne would lead to the formation of 1,4-DGB **19**.^20^ Moreover, it has been reported that reactions of an amidinato-supported disilyne with diphenylacetylene afford the corresponding 1,4-disilabenzene derivatives, albeit that the isolated 1,4-disilabenzene derivatives contain tetra-coordinated silicon atoms due to the bidentate character of the amidinato ligand.^28^ These results prompted us to examine the reaction of 1,2-DSBs and 1,2-DGBs with internal alkynes, which do not contain a H–C≡ moiety, with the objective to isolate the corresponding 1,4-DSBs and 1,4-DGBs.

**Scheme 8:**
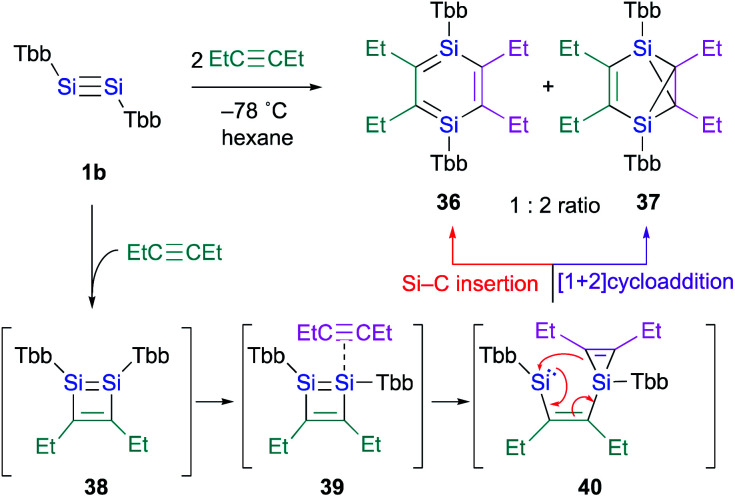
Formation of 1,4-DSB **36** in the reaction of disilyne **1b** with 3-hexyne.

In contrast to the Ge case, 1,2-disilacyclobutadiene **5** could not be isolated due to its lability on account of the intramolecular cyclization (Scheme 2b).^19^ We speculated that dialkylacetylenes, which exhibit higher nucleophilicity relative to diphenylacetylene, could potentially be more suitable for a further insertion reaction toward the transiently generated 1,2-disilacyclobutadiene to generate the corresponding 1,4-DSB without the intramolecular cyclization of the corresponding 1,2-disilacyclobutadiene intermediate as a side reaction. As expected, the reaction of disilyne **1b** with 3-hexyne resulted in the competitive formation of 1,4-DSB **36** and disilabenzvalene **37** in a 1 : 2 ratio (Scheme 8).^19^ Unfortunately, 1,4-DSB **36** is difficult to isolate from the mixture due to its lability. Interestingly, we found that **36** is photochemically converted into **37**. Although it would be feasible to think that a contamination of **37** in the reaction of disilyne **1b** with 3-hexyne could be due to the photochemical isomerization of **36**, the reaction of **1b** with 3-hexyne in the dark under otherwise identical conditions was not successful, *i.e.*, a mixture of **36** and **37** was obtained. Thus, a plausible reaction mechanism could be that shown in Scheme 8, where silirene–silylene **40**, generated *via* the orbital interaction between the π*(Si=SI) orbital of disilabutadiene intermediate **38** and the π(C≡C) orbital of 3-hexyne should be the mutual intermediate for the formation of 1,4-DSB **36** and disilabenzvalene **37**.^29^ In other words, the competitive formation of **36** and **37** should most likely be interpreted in terms of an intramolecular Si–C insertion and the intramolecular [1 + 2] cycloaddition between the silylene and the silirene moieties in **40**.

Thus, we realized that the reaction of disilynes with internal alkynes, which do not contain a H–C≡ moiety, can result in the formation of the corresponding 1,4-DSB *via* orbital interactions between the π*(Si=Si) orbital of the 1,2-disilacyclobutadiene intermediate and the π(C≡C) orbitals.

**Fig. 6:**
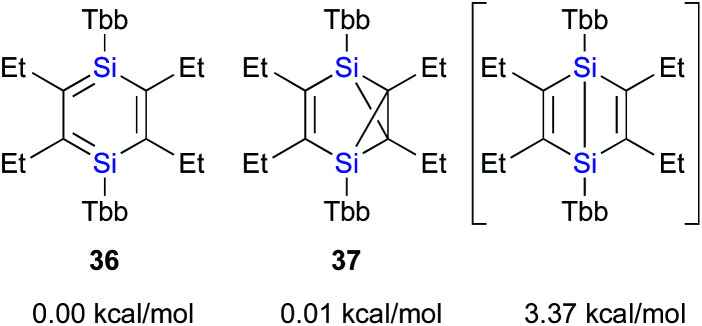
Relative energies of Tbb_2_Et_4_Si_2_C_6_ valence isomers.

The calculated potential energies of **36** and **37** are almost identical at the B3PW91-D3(BJ)/6-311G(3df,p)//B3PW91/6-311G(3df)[Si],6-31G(d)[C,H] level of theory, while that of the imaginary Dewar-type isomer is slightly higher (3.37 kcal mol^−1^) relative to that of 1,4-DSBZ **36** (Fig. 6). In contrast to the cases of the relative energies of the corresponding H_6_Si_2_C_4_ valence isomers, the energy differences between the valence isomers of the ‘real’ models are very small, suggesting that the relative stability of these valence isomers should be effectively perturbed by the electronic/steric properties of the substituents.

**Scheme 9:**
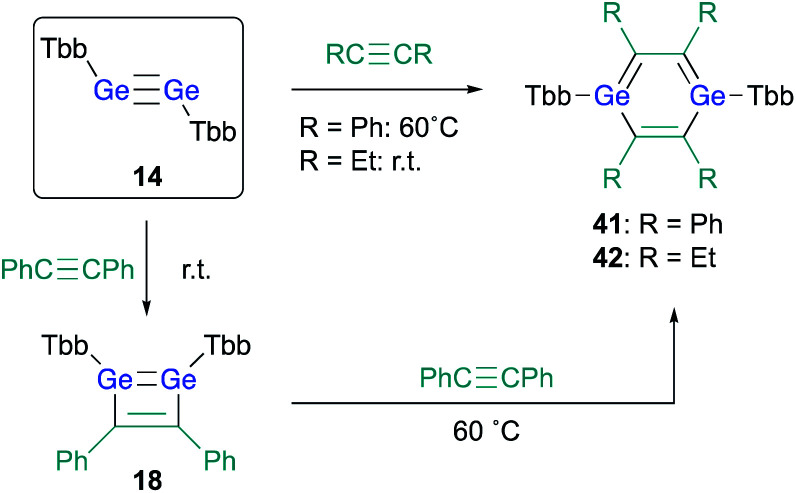
Synthesis of 1,4-DGBs **41** and **42**.

With 3,4-diphenyl-1,2-digermacyclobutadiene **18** in hand, in contrast to the Si case, the reaction of **18** with a further molecule of diphenylacetylene could be attempted in the expectation of generating the corresponding 1,4-DGB. Heating 1,2-digermabutadiene **18** in the presence of an excess of diphenylacetylene at 60 °C afforded 1,4-DGB **41** (Scheme 9).^30^ 1,4-DGB **41** should be formed *via* a mechanism similar to that for the formation of **19** (Scheme 2). Probably due to the steric demand of the Tbb and phenyl groups, a further [4 + 2] cycloaddition of **41** with diphenylacetylene does not occur, and, as a result, **41** can be isolated as a stable compound. Moreover, the reaction of digermyne **14** with 3-hexyne smoothly furnishes the corresponding 1,4-DGB **42** as the sole product, albeit that the formation of potential intermediates such as the corresponding 1,2-digermacyclobutadiene was not observed.^30^

**Scheme 10:**
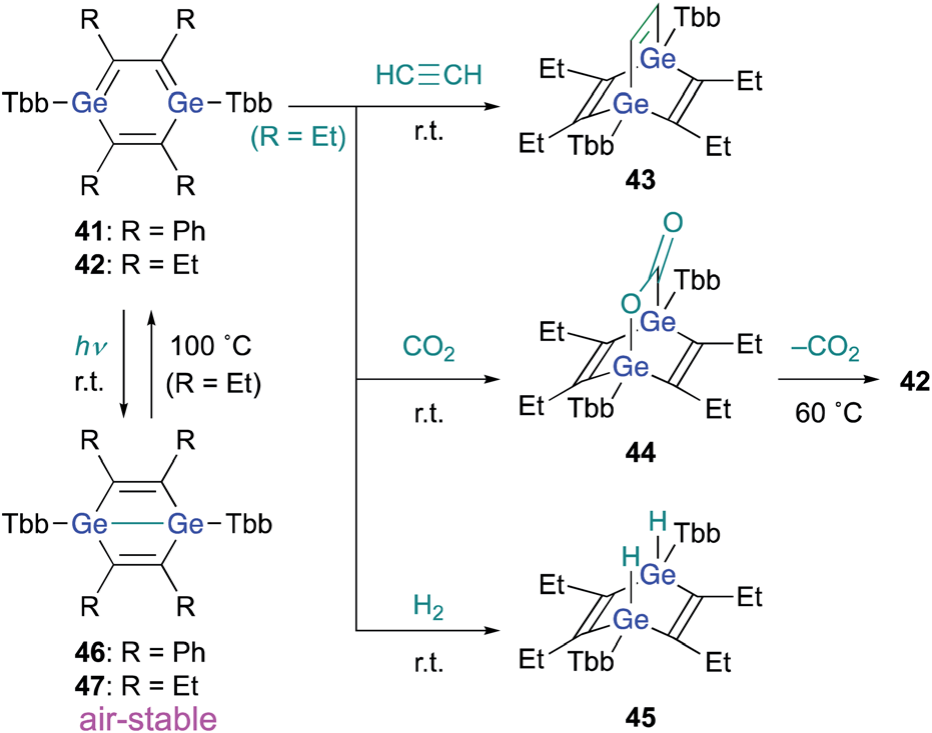
Reactions of 1,4-DGB **42** with small molecules.

When 1,4-DGB **42** was treated with acetylene, the [4 + 2] cycloadduct, *i.e.*, digermabarrelene **43** was obtained, which suggests a formation mechanism similar to that of 1,4-digermabarrelene **16** from the [4 + 2] cycloaddition of **19** with acetylene (Scheme 4). In addition, **42** was found to activate carbon dioxide (CO_2_) and molecular hydrogen (H_2_) at r.t. to give the corresponding *syn*-cycloadducts **44** and **45**, respectively. Apart from the addition of CO_2_, the addition reactions of **42** with small molecules proceed smoothly and irreversibly at r.t. to give the 1,4-adducts (Scheme 10).^30^ Interestingly, heating of **44** at 60 °C in C_6_D_6_ gave **42** with concomitant elimination of CO_2_, suggesting that the cycloaddition of 1,4-DGB **42** with CO_2_ is reversible.

Notably, 1,4-digermabenzenes **41** and **42** are photo-responsive upon exposure to LED light (410–490 nm), resulting in the quantitative formation of the corresponding digerma-Dewar-benzenes **46** and **47** (Scheme 10). The photochemical isomerization of 1,4-DGBs **46** and **47** stands in sharp contrast to that of 1,4-DSB **36**, which gave disilabenzvalene **37**; the reason for the different types of photochemical isomerization of 1,4-DSBs and 1,4-DGBs remains unclear at present. Although the photochemical isomerization of tetraphenyl-1,4-DGB **41** to digerma-Dewar-benzene **46** is thermally irreversible, tetraethyl-1,4-DGB **42** can be thermally regenerated quantitatively by heating digerma-Dewar-benzene **47** to 100 °C for 2 h in toluene. The thermal conversion of **47** can be interpreted reasonably well in terms of the relative thermodynamic energies between 1,4-DGB **42** and digerma-Dewar-benzene **47**. Theoretical calculations at the TPSSTPSS-D3(BJ)/6-311G(2d,p) level of theory showed that **47** is by 2.2 kcal mol^−1^ less stable than **42**, while **46** is by 6.9 kcal mol^−1^ more stable than **41**. It should be noted here that digerma-Dewar-benzenes **46** and **47** are inert toward air and moisture for at least two weeks, in contrast to 1,4-DGB **42**, which is highly air- and moisture-sensitive. Considering the photochemical and chemical reactivity of 1,4-DGB **42**, we attempted the activation of H_2_ with air-stable 1,4-digerma-Dewar-benzene **47**. Treatment of **47** with H_2_ (1 atm, 100 °C, toluene, 2 h) resulted in the quantitative formation of hydrogen-adduct **45**, suggesting a thermal isomerization of **47***via* intermediate **42**, which can activate H_2_. Thus, 1,4-digerma-Dewar-benzene **47** can be considered as a shelf-stable main-group-element-based catalyst for small molecules that can be activated thermally.^30^

**Fig. 7:**
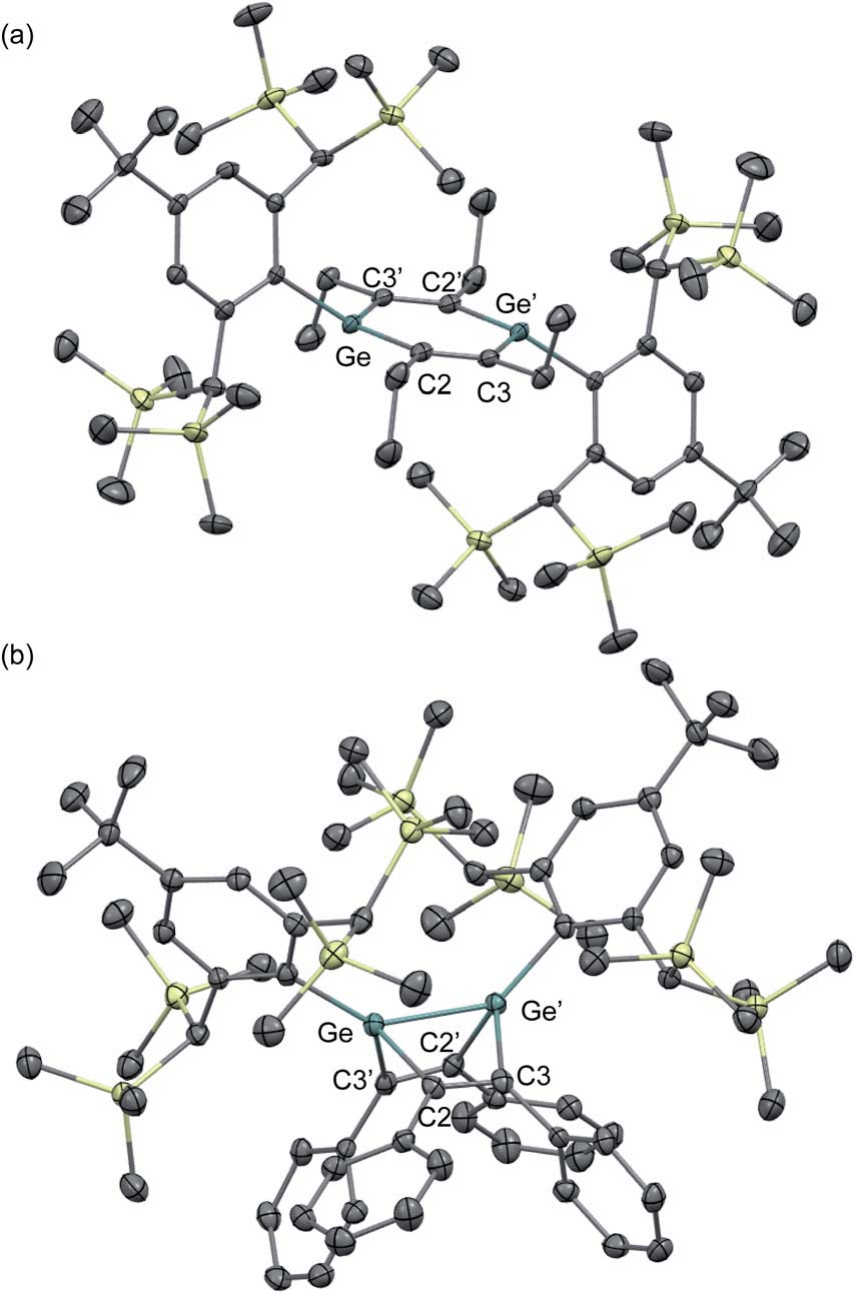
Molecular structures of (a) 1,4-DGB **42** and (b) digerma-Dewar-benzene **46** with thermal ellipsoids at 50% probability; hydrogen atoms are omitted for clarity.

The molecular structures of the obtained 1,4-DGB **42** and digerma-Dewar-benzene **46** have been determined by single-crystal XRD analyses (Fig. 7). As shown in Fig. 7, 1,4-DGB **42** exhibits a slight deviation from ideal planarity of the Ge_2_C_4_ hexagon, *i.e.*, the Ge_2_C_4_ hexagon adopts a chair-like geometry that includes a bent angle of *ca.* 10° between the Ge–C2–C3′ and C2–C3–C2′–C3′ planes. The Ge–C (1.888(3) Å) and C–C bond lengths (1.375(4) Å) in the Ge_2_C_4_ ring in **42** fall in between the corresponding double and single bond lengths, which suggests considerable π-electron delocalization over the aromatic Ge_2_C_4_ ring. In contrast, the Ge–Ge (2.3966(8) Å) and Ge–C bond lengths (2.049(4)/1.988(3) Å) in digerma-Dewar-benzene **46** are indicative of a typical Dewar-benzene structure with defined single-bond character.

In summary, as theoretically expected, the reactions of disilynes and digermynes with internal alkynes result in the formation of 1,4-DSBs and 1,4-DGBs, respectively. Interestingly, the 1,4-DSBs and 1,4-DGBs photochemically isomerize in different ways to give the corresponding disilabenzvalene and digerma-Dewar-benzene, respectively. Since the photochemical isomerization of the 1,4-DGBs is reversible upon heating, the digerma-Dewar-benzene works as a shelf-stable main-group-element-based activator for small molecules.

## 5. Conclusions

Stable 1,2-disilabenzenes (1,2-DSBs), 1,2-digermabenzenes (1,2-DGBs), and 1,4-digermabenzenes (1,4-DGBs) can be synthesized *via* the reaction of the corresponding dimetallynes with alkynes. Initially, our experimental and theoretical research was focused on a detailed understanding of the formation mechanism of the 1,2-dimetallabenzenes. The thus obtained results provided us with sufficient information to develop a strategy for the synthesis of 1,4-dimetallabenzenes. We discovered that the 1,2- and 1,4-dimetallabenzenes can be synthesized using appropriate synthetic methods, provided that sufficient kinetic stabilization is available. Their structural features suggest that these compounds exhibit considerable aromaticity, similar to that of benzene, which has been corroborated by theoretical calculations. Detailed electron-density analyses of these aromatic systems, which are currently in progress in our laboratory, can be expected to provide further important experimental insights into the aromaticity of these compounds. A fundamental investigation into the chemical properties of 1,2-DGBs revealed that a 1,2-DGB and a digermyne can work as Ge-based catalysts for the cyclotrimerization of terminal alkynes. We have thus demonstrated that a detailed fundamental investigation using a combination of experimental and theoretical techniques is able to find unique applications for the chemistry of dimetallabenzenes as main-group-element catalysts. These fundamental investigations, which combine experimental and theoretical aspects, should ultimately produce low-coordinated main-group-element compounds that serve as promising prospectives for transition-metal-free catalysts in C–C coupling reactions.

## Author contributions

The manuscript was written and checked by the author.

## Conflicts of interest

There are no conflicts to declare.
